# Supplementary material for: Asymmetric sulfonamide design enabling high-voltage sodium-ion pouch cells in wide temperature
Source: Nat Commun. 2026 Mar 24;17:4378. doi: 10.1038/s41467-026-70592-z (PMC13184103; doi:10.1038/s41467-026-70592-z)
Supplement: Supplementary file 1 — Supplementary Information [file 41467_2026_70592_MOESM1_ESM.pdf]

Supplementary Information

Asymmetric sulfonamide design enabling high-voltage sodium-ion pouch cells in wide temperature

Xinke Cui[1], Qunfang Li[1], Gang Chang[2], Wei Tang[3], Xin Huang[4], Lang Huang[5], Xue Han[6], Weijiang Xue[1,*]

[1]Center for Advancing Materials Performance from the Nanoscale (CAMP-Nano), State Key Laboratory for Mechanical Behavior of Materials, Xi'an Jiaotong University, Xi'an, Shaanxi 710049, China

[2]Instrumental Analysis Center of Xi'an Jiaotong University, Xi'an, Shaanxi 710049, China

[3]School of Chemical Engineering and Technology and National Innovation Platform (Center) for Industry-Education Integration of Energy Storage Technology, Xi'an Jiaotong University, Xi'an, Shaanxi 710049, China

[4]School of Chemistry and Chemical Engineering, Shandong University of Technology, Zibo 255049 Shandong, China

[5]Qingdao Industrial Energy Storage Research Institute, Qingdao Institute of Bioenergy and Bioprocess Technology, Chinese Academy of Sciences, Qingdao 266101, China

[6]State Key Laboratory of Metal Matrix Composites, Shanghai Jiao Tong University, Shanghai 200240, China

*Corresponding author Weijiang Xue: xuewj@xjtu.edu.cn

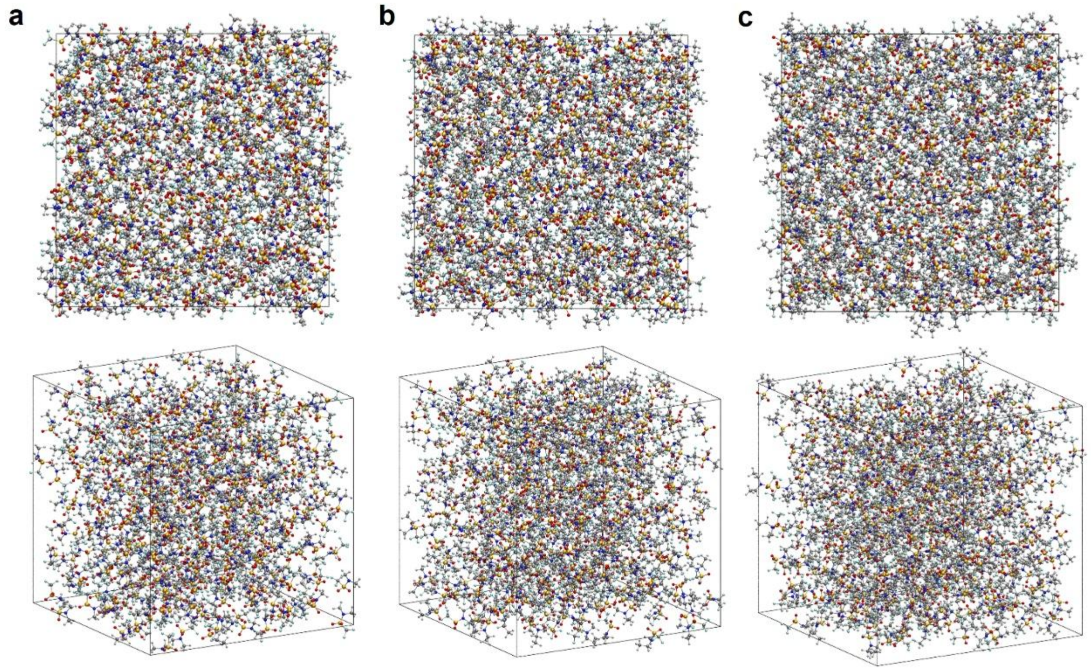

Supplementary Fig. 1 Snapshots of the MD simulation boxes. (a) DMTMSA, (b) EMTMSA, and (c) DETMSA.

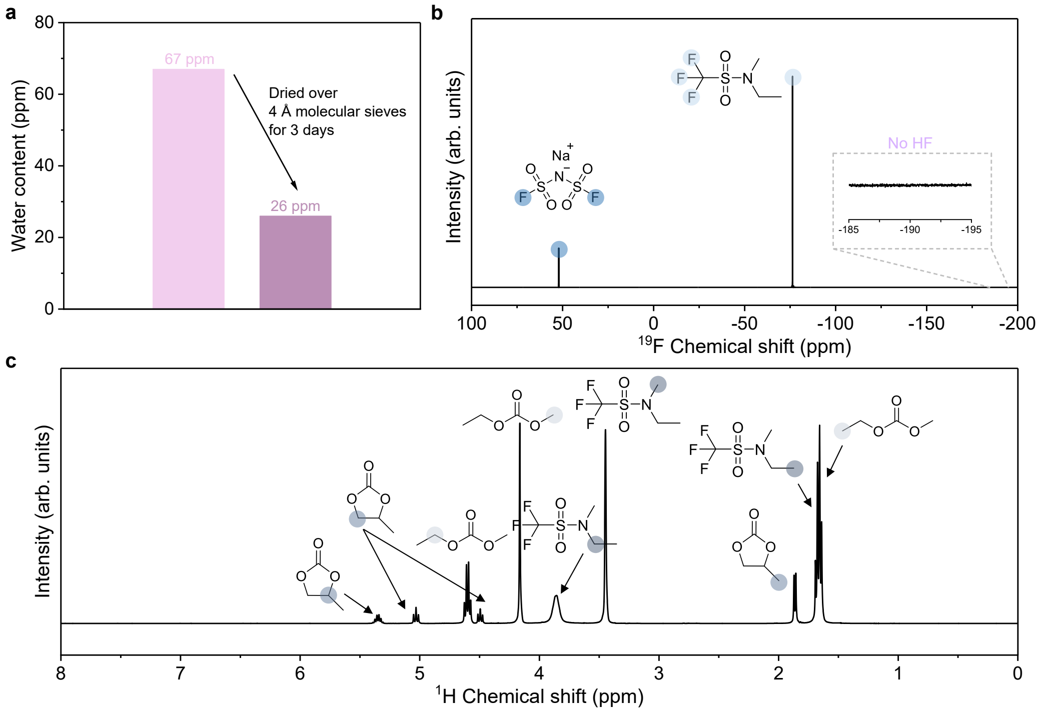

Supplementary Fig. 2 Characterizations of impurity in the EMTMSA-based electrolyte. (a) Water content of the EMTMSA-based electrolyte prepared with solvents not subjected to 4 Å molecular-sieve dehydration (left bar) and with solvents dehydrated over 4 Å molecular-sieves for 3 days (right bar). (b) ^{19}F NMR and (c) ^{1}H NMR of the dehydrated EMTMSA-based electrolyte. The molecular structures in (b) and (c), annotated with circles and arrows, indicate the peak assignments in the ^{19}F and ^{1}H NMR spectra. All observed peaks are fully assigned, and no signals appear in the HF chemical-shift region, confirming the absence of detectable HF.

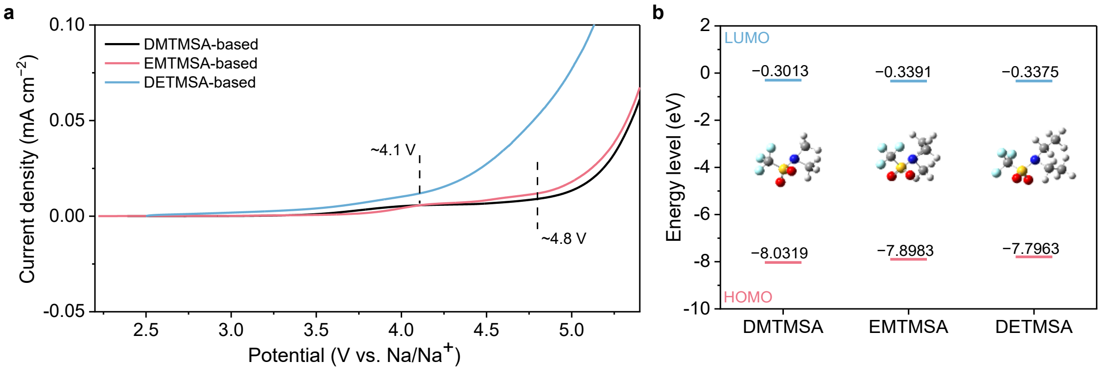

Supplementary Fig. 3 The relationship between oxidative stability and the length of the alkyl substituent of the three sulfonamide derivatives DMTMSA, EMTMSA, and DETMSA. (a) Electrochemical stability window of different electrolytes via LSV. (b) LUMO and HOMO energy levels of the DMTMSA, EMTMSA, and DETMSA calculated by DFT.

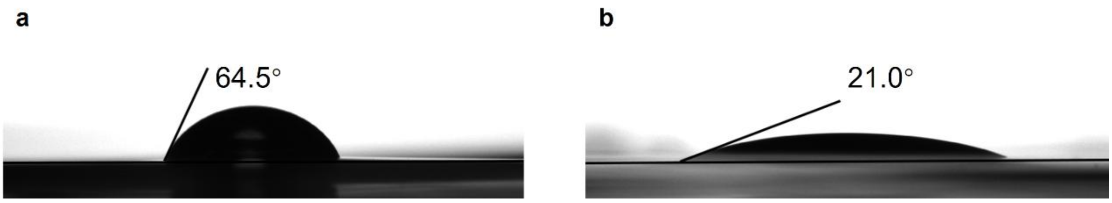

Supplementary Fig. 4 Images for measuring the contact angles on Celgard 2320 separators with the carbonate-based (a) and EMTMSA-based (b) electrolytes.

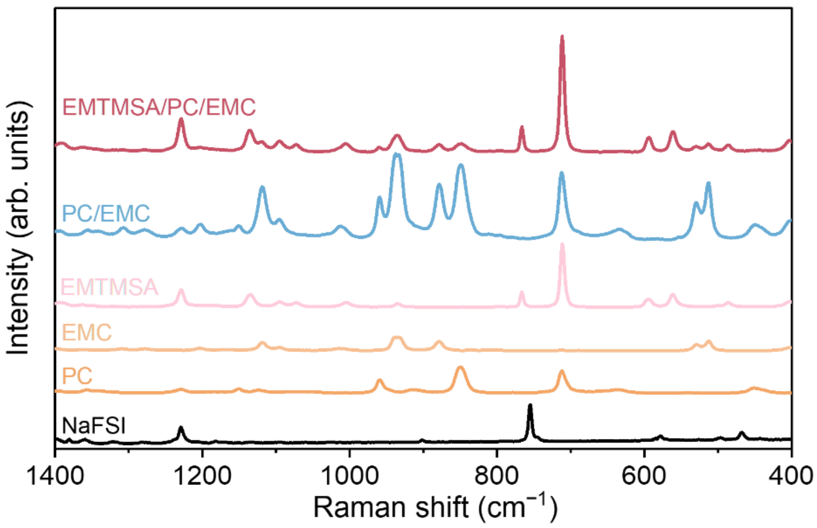

Supplementary Fig. 5 Raman spectra at 25 °C for NaFSI salt, the pure solvents (PC, EMC, and EMTMSA), and mixed solvents (PC/EMC and EMTMSA/PC/EMC).

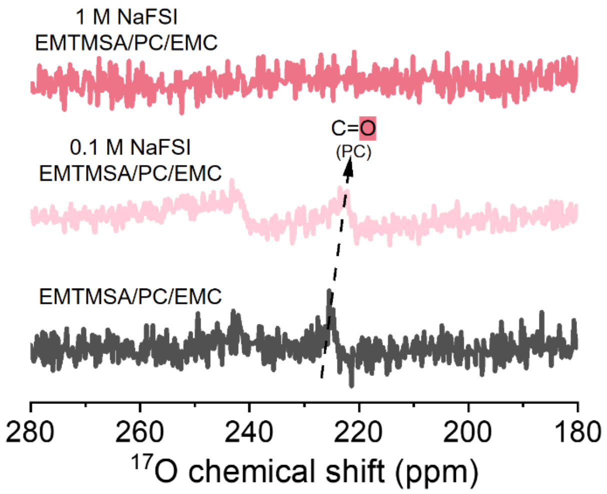

Supplementary Fig. 6 ^{17}O NMR spectra of the EMTMSA-based mixed solvent before and after incorporating 0.1 M and 1 M NaFSI.

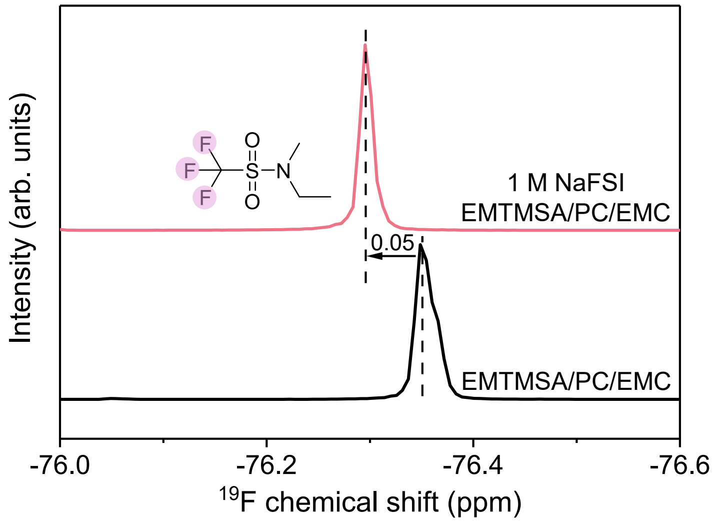

Supplementary Fig. 7 ^{19}F NMR spectra of the EMTMSA-based mixed solvent before and after incorporating 1 M NaFSI.

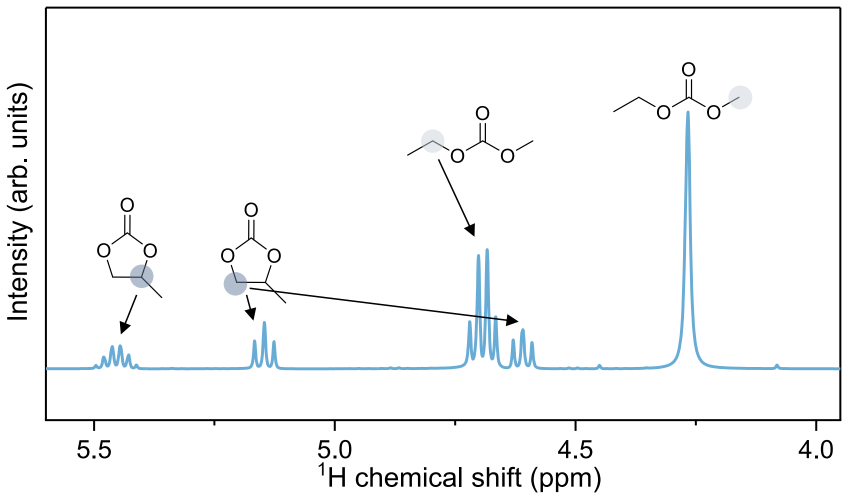

Supplementary Fig. 8 ^{1}H NMR spectra of the carbonate-based mixed solvent (PC/EMC) at room temperature.

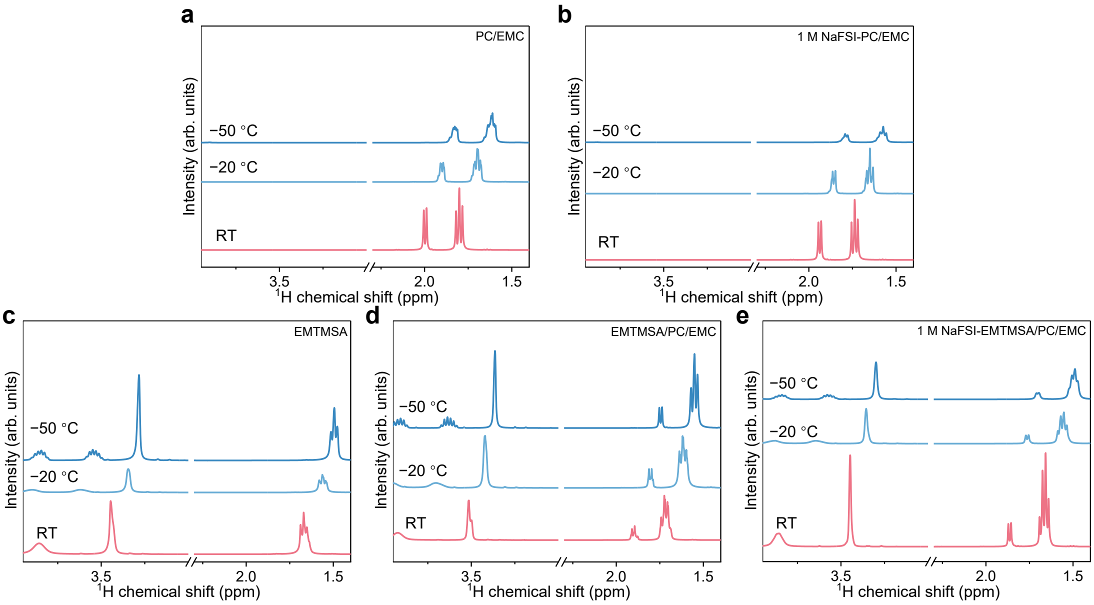

Supplementary Fig. 9 Variable-temperature ^{1}H NMR spectra. (a) PC/EMC, (b) 1 M NaFSI PC/EMC, (c) EMTMSA, (d) EMTMSA/PC/EMC, and (e) 1 M NaFSI EMTMSA/PC/EMC.

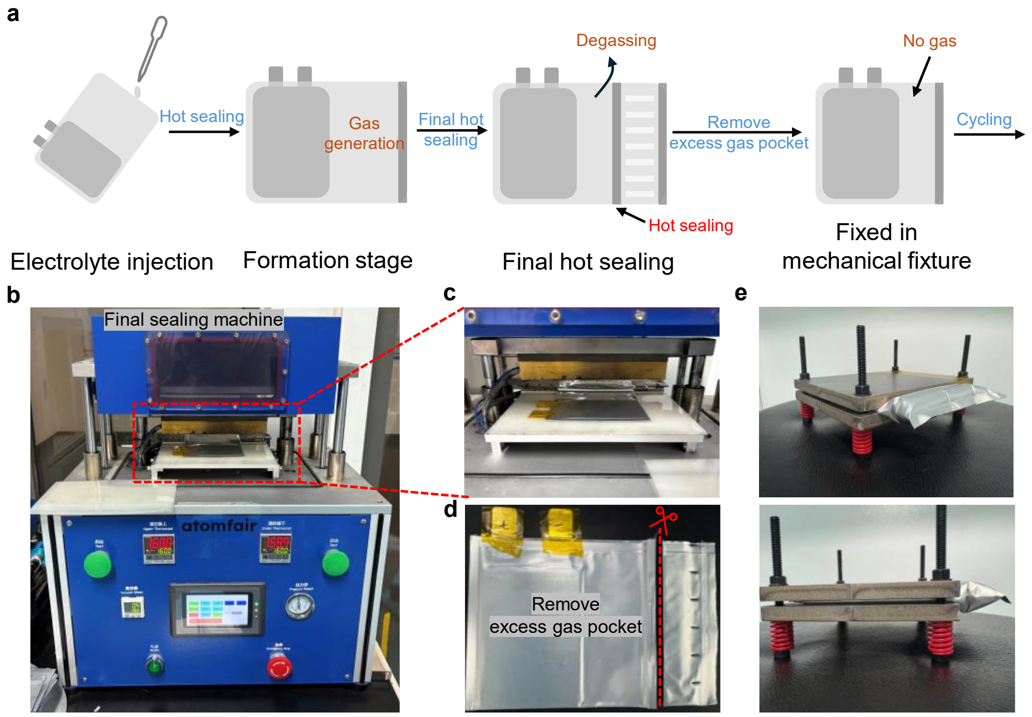

Supplementary Fig. 10 Final sealing process and the fixture for pouch cells. (a) Schematic illustration of the pouch cell final sealing and degassing processes before long-term cycling. (b) Photograph of the final sealing machine (equipped with a vacuum system and hot sealing function). (c) An enlarged photograph of the pouch cell under the hot blade of the final sealing machine. During sealing, the upper platen descends as the chamber is evacuated by a vacuum pump. Under vacuum, the edge of the gas pocket is gently punctured to release the accumulated gas, followed by hot-sealing of the residual pocket. This procedure ensures complete removal of trapped gas generated during the formation process. (d) Photograph of the pouch cell after the final sealing, with excess gas pocket removed. (e) Side-view (upper) and front-view (lower) photographs of the pouch cell mounted in the mechanical fixture after cycling. A visibly swollen gas pocket is observed along the cell edge, indicating significant gas generation during long-term cycling.

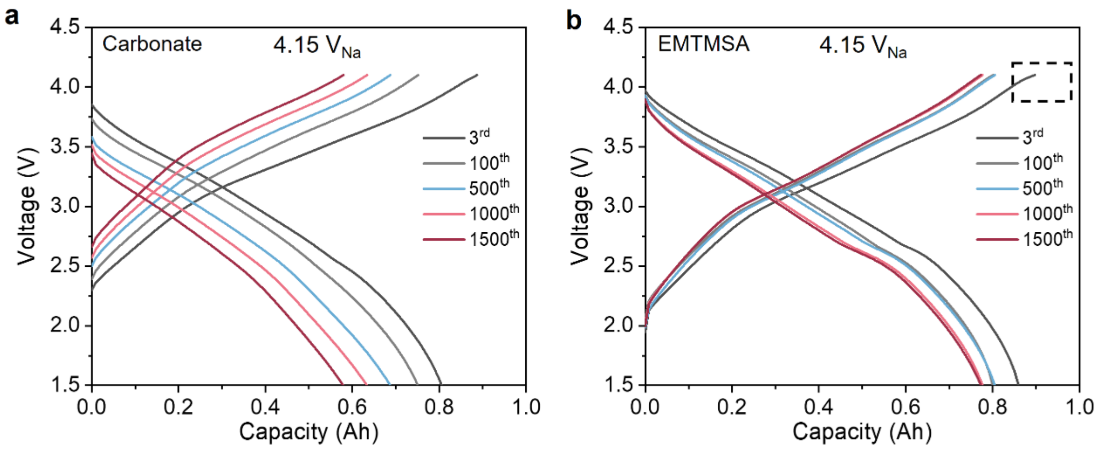

Supplementary Fig. 11 Voltage profiles with the carbonate-based (a) and EMTMSA-based (b) electrolytes at 4.15 V_{Na} cut-off voltage.

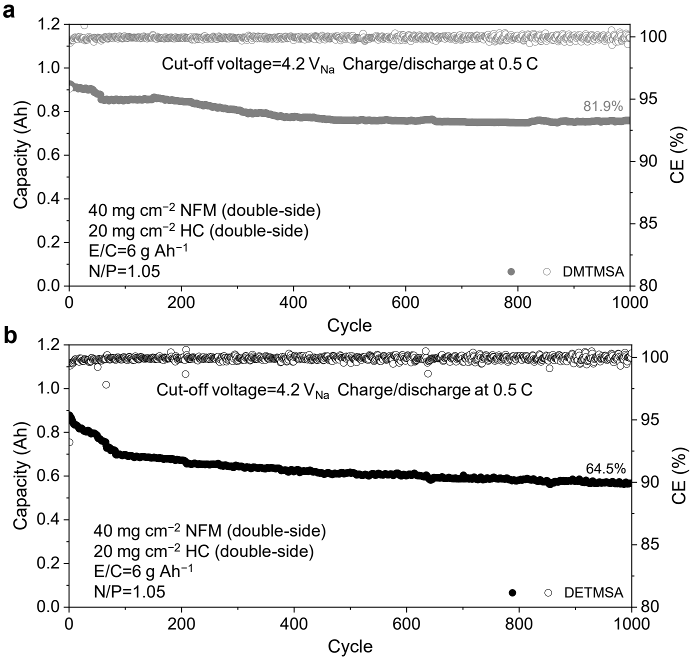

Supplementary Fig. 12 Long-term cycling performance of the pouch cells with upper cut-off voltages of 4.2 V_{Na}. (a) DMTMSA-based electrolyte. (b) DETMSA-based electrolyte. The electrolyte to capacity ratio (E/C) and negative to positive capacity ratio (N/P) of the pouch cells are indicated in the figure. The pouch cells have 8 positive electrode layers and 9 negative electrode layers. The electrochemical tests on pouch cells were conducted at a 1 A defined as 1 C rate.

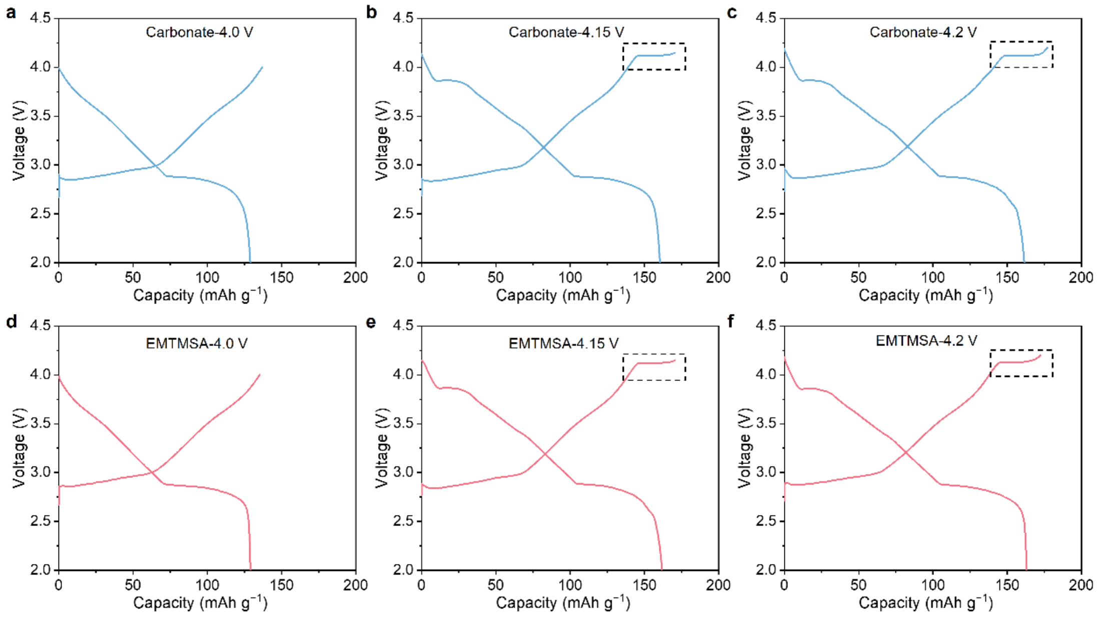

Supplementary Fig. 13 Voltage profiles of Na||NFM cells with the carbonate-based (a–c) and EMTMSA-based (d–f) electrolytes at cut-off voltages of 4.0 V, 4.15 V, and 4.2 V (vs. Na|Na^+).

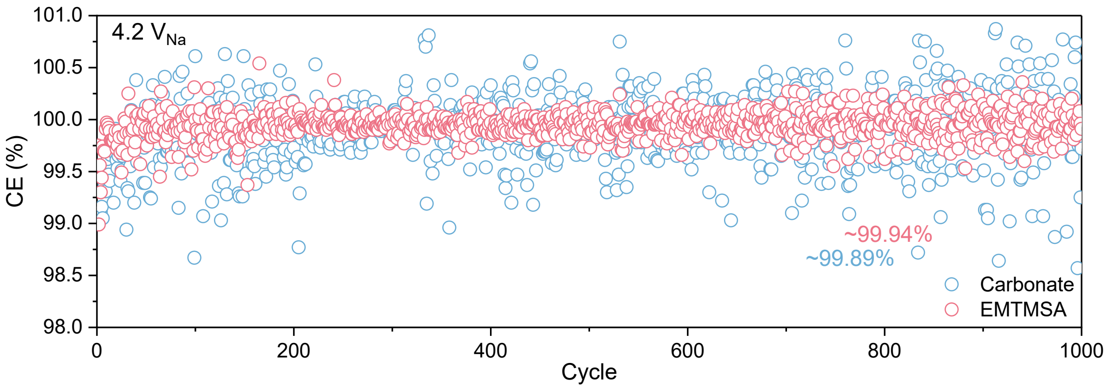

Supplementary Fig. 14 Coulombic efficiencies of the pouch cells with different electrolytes at 4.2 V_{Na} cut-off voltage.

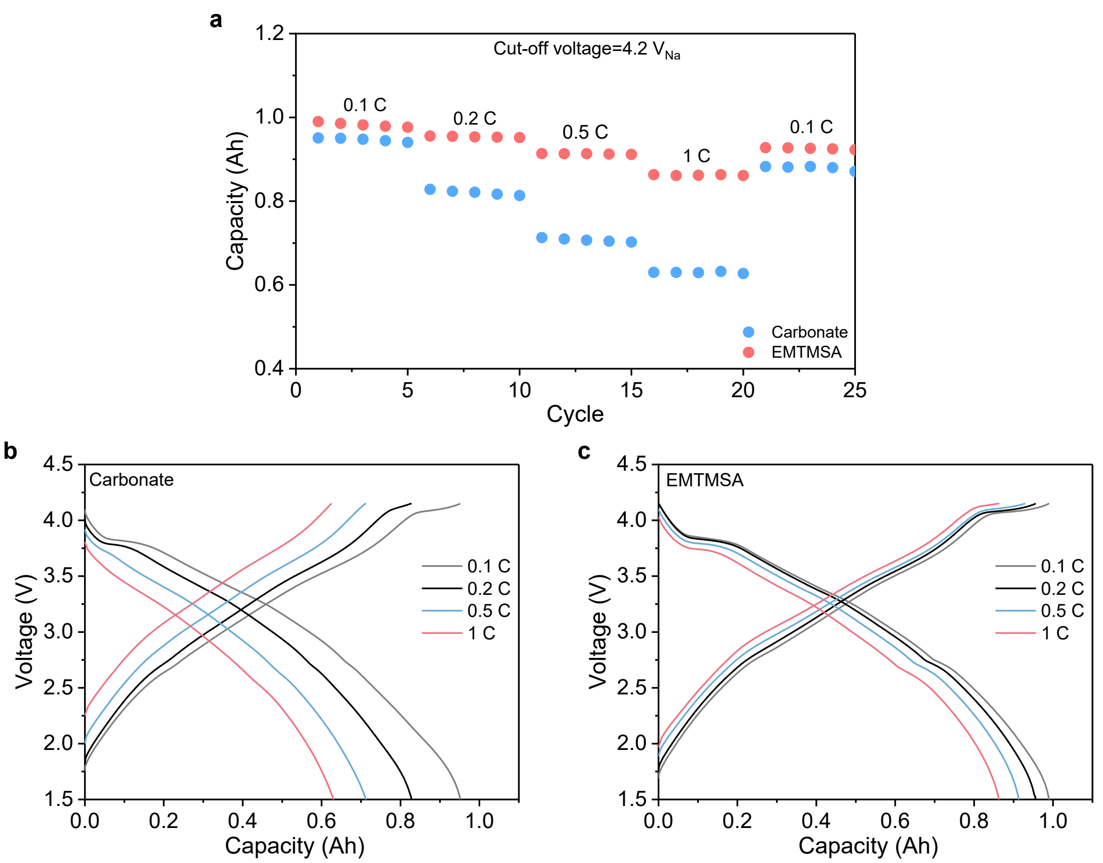

Supplementary Fig. 15 Rate performance of the pouch cells using the carbonate-based and EMTMSA-based electrolytes at different rates and upper cut-off voltage of 4.2 V_{Na}. (a) Comparison of discharge capacity of the pouch cells at different rates. Corresponding voltage profiles for the (b) carbonate-based and (c) EMTMSA-based electrolytes.

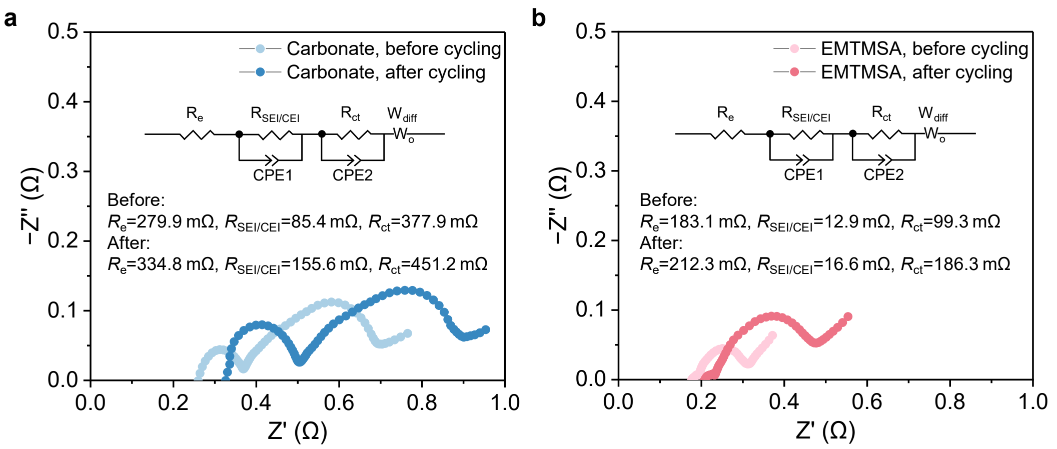

Supplementary Fig. 16 The Nyquist plots, along with the equivalent circuit model and parameters (insets), fitted from EIS analysis on the HC||NFM pouch cells using the (a) carbonate-based and (b) EMTMSA-based electrolytes before and after long-term cycling at 4.2 V_{Na}.

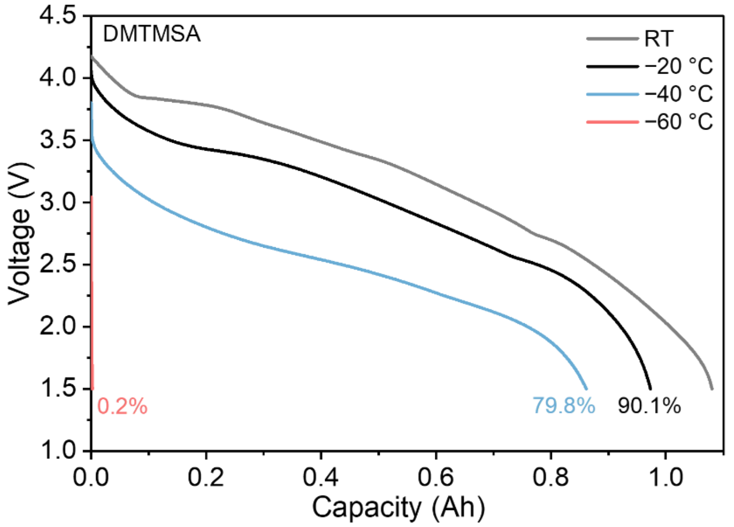

Supplementary Fig. 17 Voltage profiles of the pouch cell with the DMTMSA-based electrolyte during discharging at 25 °C, −20 °C, −40 °C, and −60 °C.

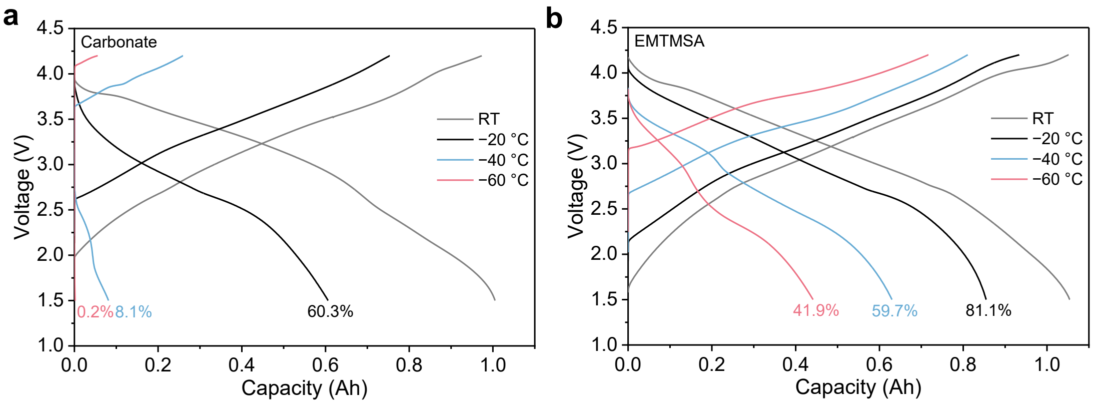

Supplementary Fig. 18 Voltage profiles of pouch cells with the (a) carbonate-based and (b) EMTMSA-based electrolytes during charge/discharge at a rate of 0.01 C, measured at 25 °C, −20 °C, −40 °C, and −60 °C.

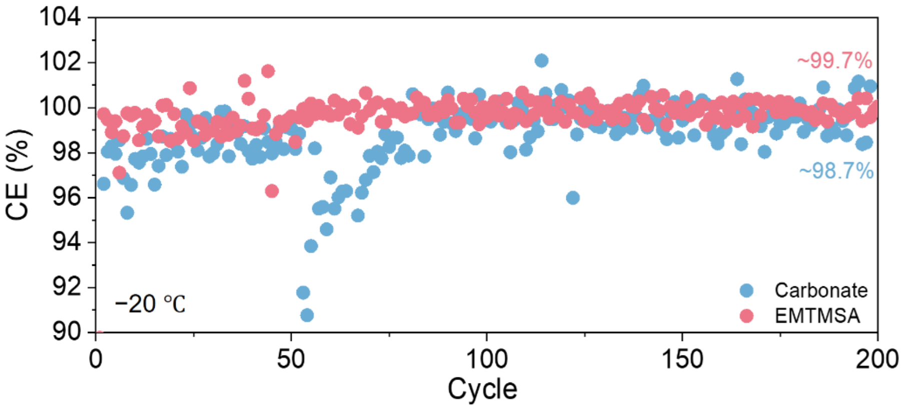

Supplementary Fig. 19 Coulombic efficiencies of HC||NFM pouch cells as a function of cycle number with different electrolytes at −20 °C.

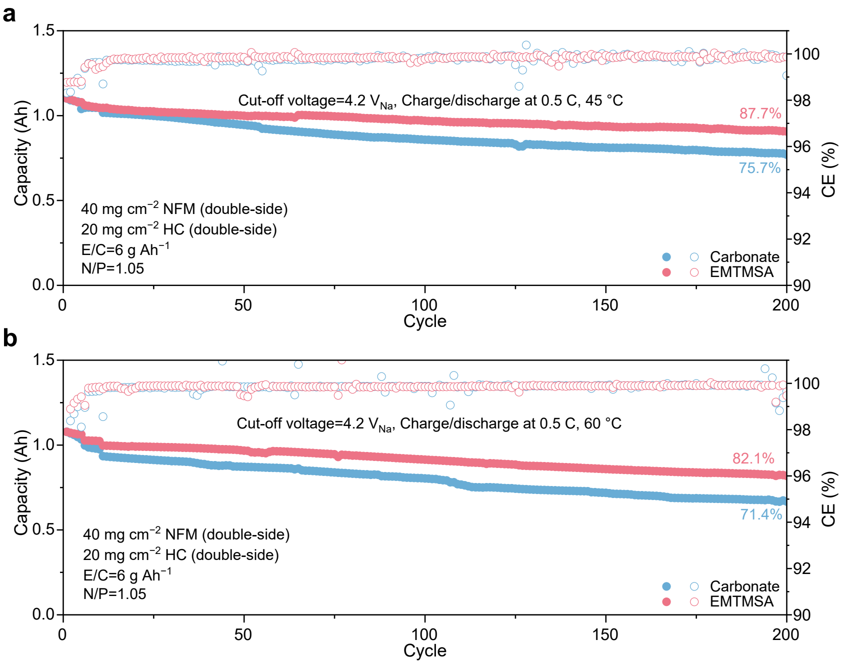

Supplementary Fig. 20 Cycling performance of pouch cells with the carbonate-based and EMTMSA-based electrolytes at high temperatures with an upper cut-off voltage of 4.2 V_{Na} at 0.5 C. (a) 45 °C. (b) 60 °C. The electrolyte to capacity ratio (E/C) and negative to positive capacity ratio (N/P) of the pouch cells are indicated in the figure. The pouch cells have 8 positive electrode layers and 9 negative electrode layers. The electrochemical tests on pouch cells were conducted at a 1 A defined as 1 C rate.

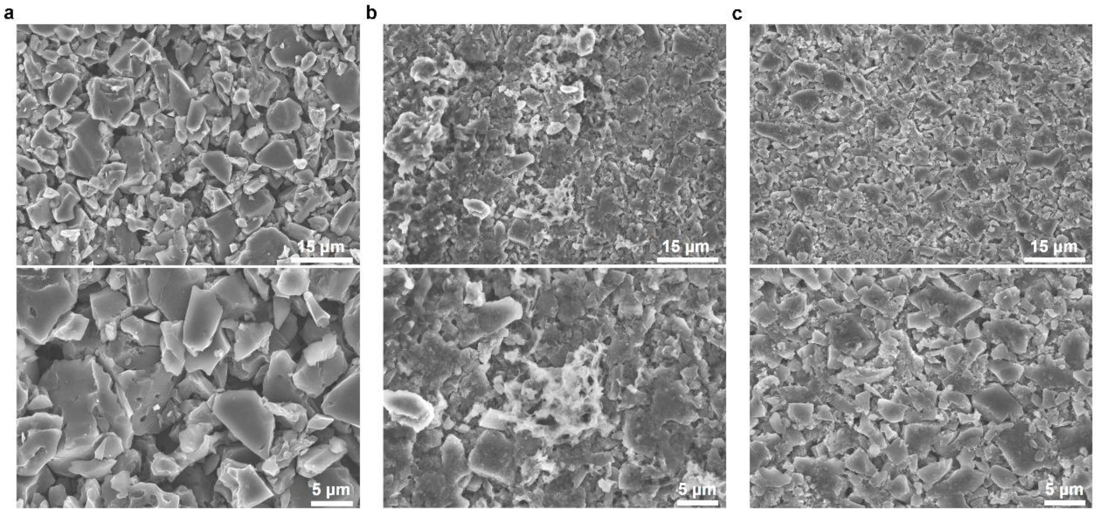

Supplementary Fig. 21 SEM images of HC electrodes after cycling. (a) Pristine electrode, electrode cycled in the (b) carbonate-based, and (c) EMTMSA-based electrolytes.

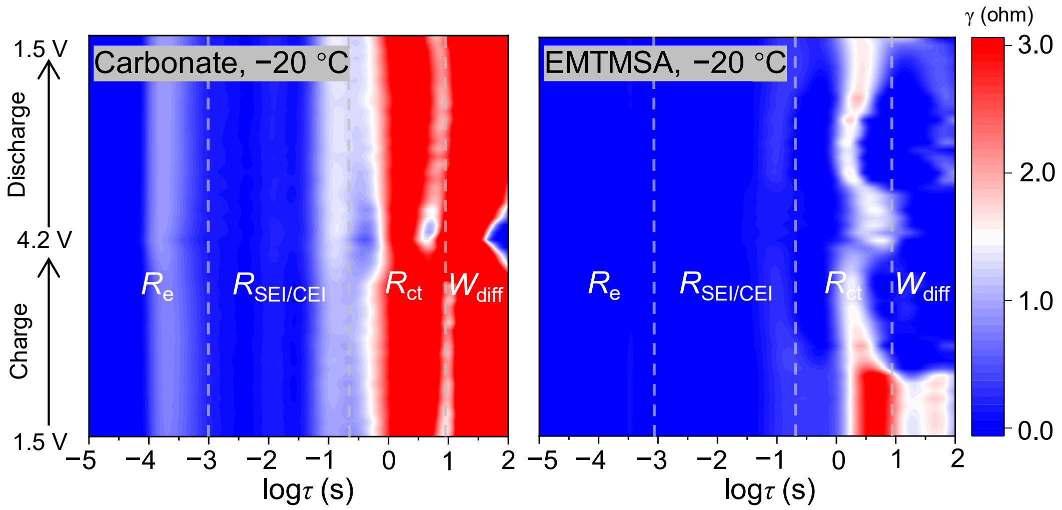

Supplementary Fig. 22 In situ distribution of relaxation times data of the pouch cells with carbonate-based (left) and EMTMSA-based (right) electrolytes at −20 °C upon charging and discharging between 1.5 V to 4.2 V. The four distinct regions in DRT data are attributed to R_e (electrolyte resistance), $R_{SEI/CEI}$ (SEI and CEI resistance), R_{ct} (charge transfer resistance), and W_{diff} (solid-state diffusion). Blue and red colors correspond to the minimum and maximum intensities respectively.

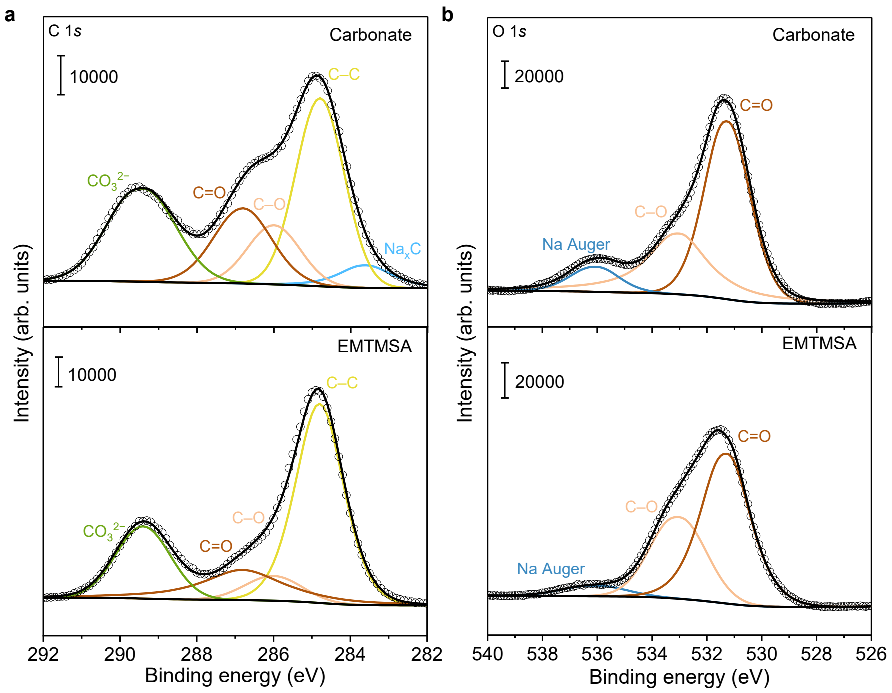

Supplementary Fig. 23 XPS analysis for the SEIs formed on HC with different electrolytes. (a) C 1*s* spectra of the SEIs formed on HC. (b) O 1*s* spectra of the SEIs formed on HC. The intensity scales on the y-axis are indicated.

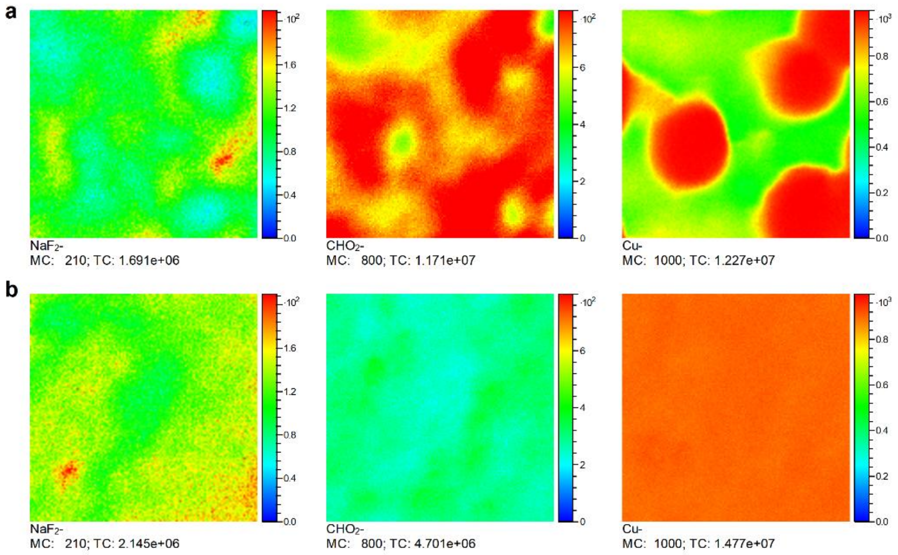

Supplementary Fig. 24 2D renderings of NaF_2^-, CHO_2^-, and Cu^- signals collected from TOF-SIMS for the SEI formed on Cu current collectors with the carbonate-based (a) and EMTMSA-based (b) electrolytes. Blue and red colors correspond to the minimum and maximum intensities respectively.

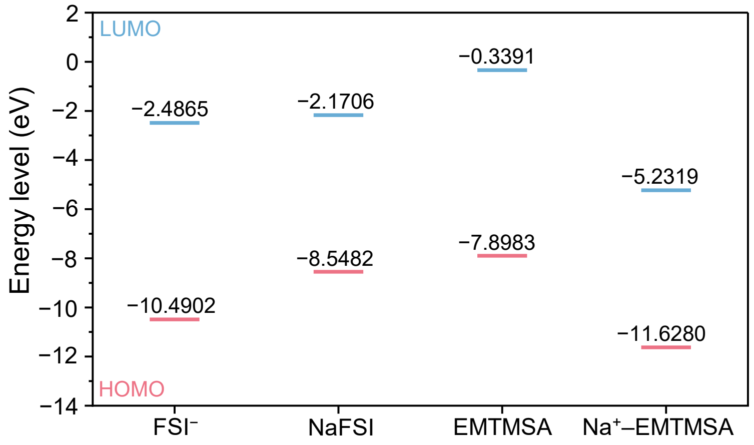

Supplementary Fig. 25 The LUMO and HOMO energy levels of the FSI^-, NaFSI, EMTMSA, and Na^+–EMTMSA calculated by DFT calculations. The trends in energy levels imply that under such solvation conditions, oxidative decomposition preferentially occurs on FSI^- while reductive decomposition is more likely on Na^+–EMTMSA, collectively leading to the formation of inorganic- and F-containing interphases in the EMTMSA-based electrolyte.

Supplementary Table 1. Physicochemical parameters of the EMTMSA solvent.

	Melting point	Flash point	Boiling point	Dielectric constant	Viscosity
EMTMSA	−86 °C	170 °C	155 °C	8.21	1.8 mPa s

Supplementary Table 2. Cell parameters of the 1 Ah HC||NFM pouch cell.

Positive electrode	Active material	NFM
	Active material percentage (wt%)	95.3%
	Areal loading (both side, mg cm^{-2})	40
	Length (mm)*width (mm)	60*80
Negative electrode	Active material	HC
	Active material percentage (wt%)	94.5%
	Areal loading (both side, mg cm^{-2})	20.06
	Length (mm)*width (mm)	63*84
Layer of positive electrodes		8
Layer of negative electrodes		9
Negative to positive capacity ratio		1.05
Thickness of separator (μm)		12
Electrolyte to capacity ratio (g Ah^{-1})		6

Supplementary Table 3. Error values of EIS analysis for the HC||NFM pouch cells before and after long-term cycling at 4.2 V_{Na} in different electrolytes.

	Carbonate before cycling	Carbonate after cycling	EMTMSA before cycling	EMTMSA after cycling
R_e (Error)	0.00237	0.00382	0.00135	0.00218
R_e (Error%)	0.86970	1.51390	0.51601	0.79978
$R_{SEI/CEI}$ (Error)	0.00355	0.00607	0.00128	0.00109
$R_{SEI/CEI}$ (Error%)	4.15940	3.90060	5.61100	6.13540
R_{ct} (Error)	0.08311	0.01320	0.00378	0.00581
R_{ct} (Error%)	3.19240	2.97963	3.80030	3.12020

Supplementary Table 4. Comparison of our work with other low-temperature electrolyte studies on batteries based on layered oxide positive electrodes.

Electrolyte	Negative electrode‖Positive electrode	Cell configuration	Voltage range	Capacity retention
**1 M NaFSI-EMTMSA/PC/EMC (our work)**	**HC‖NaNFM**	**pouch cell**	**1.5–4.2 V**	**69.8% (−60 °C)** **42.3% (−70 °C)**
1 M $NaPF_6$-EC/PC/DEC+5% FEC+3% ADN[1]	HC‖NaNFM	pouch cell	1.5–3.9 V	76.5% (−20 °C)
0.3 M $NaPF_6$-EC/PC[2]	AC‖NaCNFM	coin cell	1.0–4.0 V	84.0% (−30 °C)
1 M $NaPF_6$-PC/EMC+2% FEC[3]	HC‖NaNFM	pouch cell	1.5–3.8 V	52.2% (−40 °C)
1 M $NaPF_6$-G2/THF[4]	HC‖NaNCM	coin cell	1.5–3.65 V	70.4% (−40 °C)
1 M $NaPF_6$-EC/PC/DEC+5% FEC+2% FPPN[5]	HC‖NaNFM	pouch cell	2.0–4.0 V	58.9% (−40 °C)
0.5 M $NaPF_6$-G2[6]	HC‖LMNM‘T	coin cell	2.2–4.15 V	89.9% (−30 °C)
0.8 M $NaPF_6$-EC/EMC +1% NaDFP[7]	HC‖NaNFM	pouch cell	1.5–3.8 V	79.2% (−30 °C)
1 M NaFSI-AN/OTE[8]	HC‖NaNFM	pouch cell	1.5–3.85 V	56.4% (−60 °C)

Supplementary References

1. Song, X. *et al.* The effects of the functional electrolyte additive on the cathode material $Na_{0.76}Ni_{0.3}Fe_{0.4}Mn_{0.3}O_2$ for sodium-ion batteries. *Electrochim. Acta* **281**, 370–377 (2018).
2. Li, Y. *et al.* Ultralow-concentration electrolyte for Na-ion batteries. *ACS Energy Lett.* **5**, 1156–1158 (2020).
3. Che, H. *et al.* Engineering optimization approach of nonaqueous electrolyte for sodium ion battery with long cycle life and safety. *Green Energy Environ.* **6**, 212–219 (2021).
4. Yang, C. *et al.* Entropy-driven solvation toward low-temperature sodium-ion batteries with temperature-adaptive feature. *Adv. Mater.* **35**, 2301817 (2023).
5. Liao, Y. *et al.* Pentafluoro(phenoxy)cyclotriphosphazene stabilizes electrode/electrolyte interfaces for sodium-ion pouch cells of 145 Wh Kg^{-1}. *Adv. Mater.* **36**, 2312287 (2024).
6. Feng, Y. *et al.* Monolithic interphase enables fast kinetics for high-performance sodium-ion batteries at subzero temperature. *Angew. Chem. Int. Ed.* **63**, e202403585 (2024).
7. Cai, J. *et al.* A dual-functional electrolyte additive for stabilizing the solid electrolyte interphase and solvation structure to enable pouch sodium ion batteries with high performance at a wide temperature range from −30 °C to 60 °C. *Chem. Eng. J.* **491**, 151949 (2024).
8. Liu, G. *et al.* Practical and versatile sodium-ion batteries realized with nitrile-based electrolytes. *Adv. Energy Mater.* **15**, 2405319 (2025).
