# Supplementary material for: Asymmetric sulfonamide design enabling high-voltage sodium-ion pouch cells in wide temperature
Source: Nat Commun. 2026 Mar 24;17:4378. doi: 10.1038/s41467-026-70592-z (PMC13184103; doi:10.1038/s41467-026-70592-z)
Supplement: Supplementary file 2 — Description of Additional Supplementary Files [file 41467_2026_70592_MOESM2_ESM.pdf]

Description of Additional Supplementary Files

Supplementary Data 1: Atomic coordinates used in the MD simulations to compute van der Waals and Coulomb forces for the three sulfonamide molecules (DMTMSA, EMTMSA, and DETMSA) at different low temperatures.

Supplementary Data 2: Atomic coordinates used in the DFT to compute LUMO and HOMO energy levels.

Supplementary Movie 1: Ignition test of carbonate-based electrolyte: the liquid ignites and burns when exposed to a flame.

Supplementary Movie 2: Ignition test of EMTMSA-based electrolyte: the liquid shows no combustion, demonstrating its non-flammable property.
